# Salivary Pellicle Formed on Dental Composites Evaluated by Mass Spectrometry—An In Situ Study

**DOI:** 10.3390/molecules28196804

**Published:** 2023-09-26

**Authors:** Markus Reise, Stefan Kranz, Markus Heyder, Julius Beck, Christian Roth, André Guellmar, Ferdinand von Eggeling, Ulrich Schubert, Bettina Löffler, Bernd Sigusch

**Affiliations:** 1 Department of Conservative Dentistry and Periodontology, Jena University Hospital, Friedrich-Schiller University, An der alten Post 4, 07743 Jena, Germany; markus.reise@med.uni-jena.de (M.R.); markus.heyder@med.uni-jena.de (M.H.); julius.beck@med.uni-jena.de (J.B.); andre.guellmar@med.uni-jena.de (A.G.); bernd.w.sigusch@med.uni-jena.de (B.S.); 2 Department of Otorhinolaryngology, Jena University Hospital, Am Klinikum 1, 07747 Jena, Germany; feggeling@med.uni-jena.de; 3 Institute of Organic Chemistry and Macromolecular Chemistry, Friedrich Schiller University Jena, Humboldtstrasse 10, 07743 Jena, Germany; ulrich.schubert@uni-jena.de; 4 Institute of Medical Microbiology, Jena University Hospital, Friedrich-Schiller University, Erlanger Allee 101, 07747 Jena, Germany; bettina.loeffler@med.uni-jena.de

**Keywords:** dental materials, dental pellicle, enamel pellicle, proteomic profile, restoratives, resin-based composites, salivary acquired pellicle, salivary proteins

## Abstract

(1) Background: In the oral environment, sound enamel and dental restorative materials are immediately covered by a pellicle layer, which enables bacteria to attach. For the development of new materials with repellent surface functions, information on the formation and maturation of salivary pellicles is crucial. Therefore, the present in situ study aimed to investigate the proteomic profile of salivary pellicles formed on different dental composites. (2) Methods: Light-cured composite and bovine enamel samples (controls) were exposed to the oral cavity for 30, 90, and 120 min. All samples were subjected to optical and mechanical profilometry, as well as SEM surface evaluation. Acquired pellicles and unstimulated whole saliva samples were analyzed by SELDI–TOF–MS. The significance was determined by the generalized estimation equation and the post-hoc bonferroni adjustment. (3) Results: SEM revealed the formation of homogeneous pellicles on all test and control surfaces. Profilometry showed that composite surfaces tend to be of higher roughness compared to enamel. SELDI–TOF–MS detected up to 102 different proteins in the saliva samples and up to 46 proteins in the pellicle. Significant differences among 14 pellicle proteins were found between the composite materials and the controls. (4) Conclusions: Pellicle formation was material- and time-dependent. Proteins differed among the composites and to the control.

## 1. Introduction

After a professional dental cleaning, sound enamel surfaces and restorative materials are immediately covered by a thick layer of proteins, glycoproteins, carbohydrates, and lipids that mainly origin from the saliva and sulcus fluid [1,2,3]. This layer is defined as acquired enamel pellicle and subjected to a time-dependent process of maturation [4,5]. Within 30 to 60 min, the pellicle appears as a loosely formed, globular or granular structure followed by an increase in thickness. After a 2 h maturation period and depending on the intraoral location, the pellicle layer can reach values of between 20 and 700 nm and might double within 24 h [6,7].

One important function of the pellicle layer is to prevent attrition and abrasion of the tooth’s hard tissues by acting as a lubricant [2,3,5,8]. In addition, the acquired pellicle is able to control dental erosion by modulating calcium and phosphate concentrations on the tooth surface [1,5,9]. Furthermore, due to the incorporation of components from the host immune system, such as agglutinins, complement factors, secretory immunoglobulins, lysozyme, lactoferrin, and peroxidases, the acquired enamel pellicle also acts as an antimicrobial barrier [10,11,12,13].

Besides carbohydrates and lipids, proteins are the main component of an acquired salivary pellicle. Overall, up to 1000 different proteins can be detected [14]. Out of these, phosphoproteins are the most common. Other proteins that are especially rich in glutamic acid, glycine, alanine, serine, and proline are also usually present in high numbers [5,13,14,15,16,17].

Up to now, significant differences in the ultrastructural appearance of in situ-formed pellicle layers on enamel and restorative materials such as ceramics, cement, resin-based composites, or titanium are still controversially discussed [14,18,19]. While some authors report variations in the accumulation of specific proteins such as secretory immunoglobulin A, immunoglobulin G, peroxidases, thiocyanate, and lysozyme, others did not observe any significant differences [15,20,21,22,23,24,25,26].

As already known, a mature acquired pellicle also provides specific receptors for oral bacteria to attach [1,26]. This will cause a highly specific and irreversible attachment of bacterial cells to the dental surface. Thus, the acquired salivary pellicle acts as a conditioning film, controlling the initial steps of biofilm formation [27,28]. Although the available data suggest that microbial succession on dental materials is similar to that on a sound enamel, differences in the abundance of relevant species may still exist [7]. Observations have also shown that conventional dental materials such as resin-based composites are sometimes afflicted by a rather extensive formation of bacterial biofilms, even under healthy oral conditions [17].

So far, there are currently no studies available that report an influence of resin-based composites on the pellicle-binding behavior. In this context, novel and improved materials with bio-repelling functions and easy-to-clean surface characteristics are highly required [29].

Especially for resin-based composites, information upon pellicle formation and maturation is still rare. Thus, new data about these issues could contribute to the development of innovative anti-biofilm surface strategies. Therefore, the present study aimed to investigate the proteomic profile of salivary pellicle layers formed on modern dental composites after a time-dependent exposure to the oral cavity.

## 2. Results

### 2.1. SEM Evaluation

**Figure 1:**
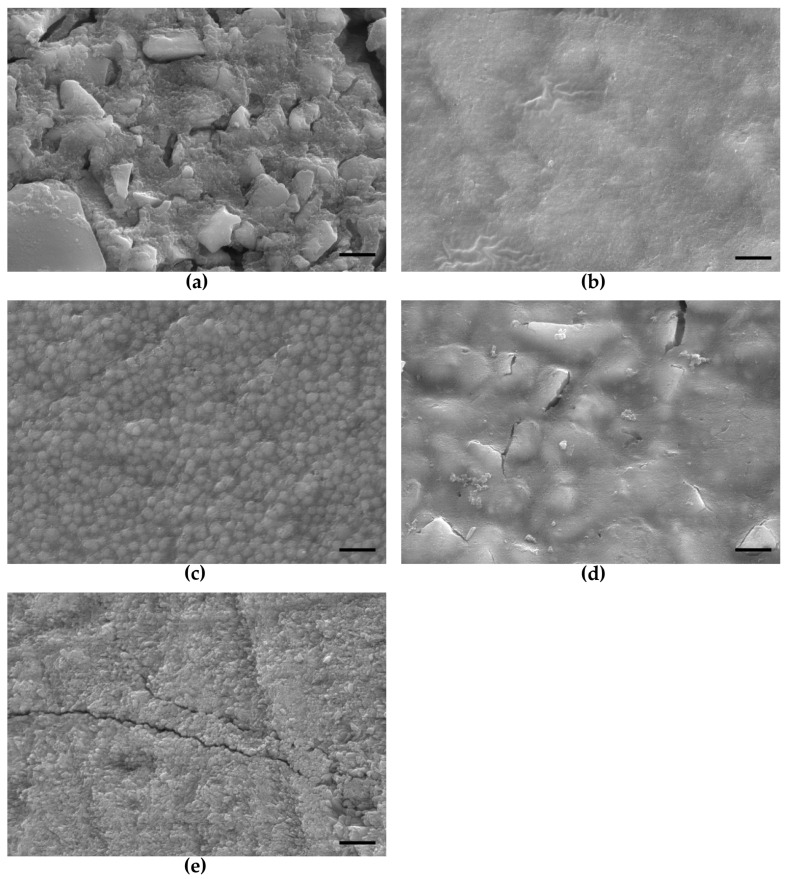
SEM surface images of the respective dental composites and the bovine enamel control prior to oral exposure: (**a**) Venus Diamond; (**b**) GrandioSO; (**c**) Estelite Σ Quick; (**d**) Dyract eXtra; (**e**) Bovine enamel.

Representative images of all native composite surfaces and of the bovine enamel control prior to oral exposure are shown in Figure 1.

Depending on the material composition (filler size and content), specific differences in the surface topography were observed. An especially rough surface with a pronounced exposure of filler particles was analyzed for Venus^®^ Diamond and for Dyract^®^ eXtra. Estelite Σ Quick showed a homogeneous and smooth surface appearance with spherical submicron-sized filler particles uniformly embedded into the resin matrix. A similar surface topography was also found for GrandioSO with glass-ceramic fillers well-integrated into the resin matrix.

The bovine enamel control displayed a homogeneous surface with unregular depressions, artificial cracks, and exposed enamel prisms.

**Figure 2:**
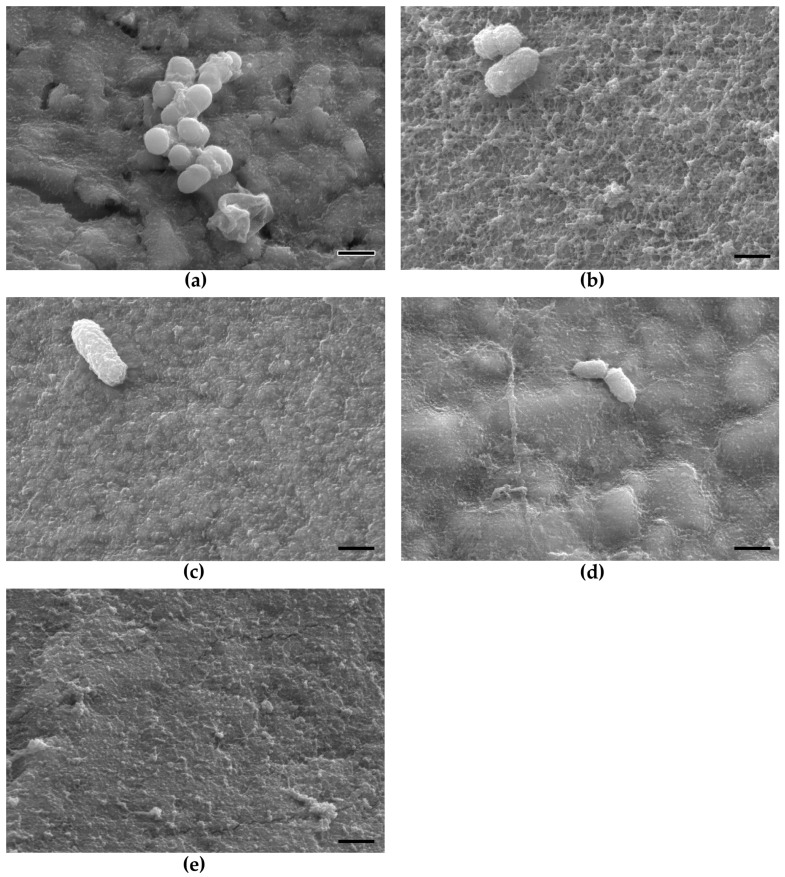
SEM images after exposure of the composite materials and the enamel control to the oral environment for 120 min. The material surface is covered by a uniform pellicle with adhered oral bacteria: (**a**) Venus Diamond; (**b**) GrandioSO; (**c**) Estelite Σ Quick; (**d**) Dyract eXtra; (**e**) Bovine enamel. Pellicle formation enabled early bacterial species to attach.

In Figure 2, SEM images of all surfaces are shown after an exposure to the oral environment for 120 min. Each material surface is uniformly covered by an acquired pellicle. In the case of Venus^®^ Diamond and Dyract^®^ eXtra, the distinct rough surface topography can still be recognized. The pellicle layer formed on the enamel control samples appeared in a uniform net-like structure. Furthermore, adherent oral bacteria were also present in the SEM images.

### 2.2. Profilometry

**Figure 3:**
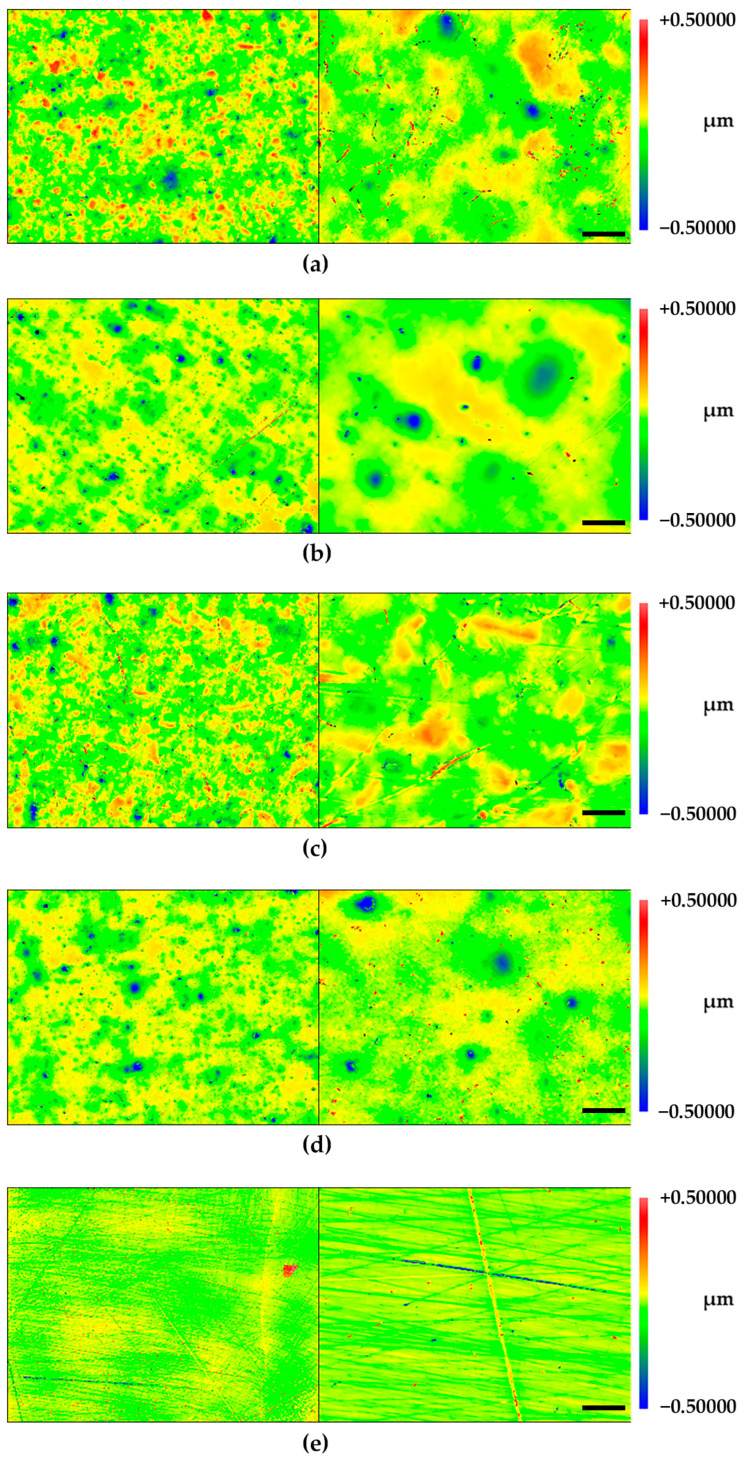
White-light interferometry of the native composite and enamel surfaces: (**a**) Venus^®^ Diamond; (**b**) GrandioSO; (**c**) Estelite Σ Quick; (**d**) Dyract^®^ eXtra; (**e**) Bovine enamel control. Left vertical row in 10× magnification; right vertical row in 50× magnification.

In Figure 3, the results of the white light interferometry are presented. The findings are partly supported by observations obtained in the SEM evaluation.

The determined Ra values did not show any significant roughness differences among the tested restorative materials (68–80 nm). In comparison, the enamel controls displayed lower Ra values (Table 1).

### 2.3. SELDI–TOF–MS

An analyzation of the samples using SELDI–TOF–MS caused a great diversity of different signals. Out of these, only those with intensities > 1 were subjected to detailed examination. Results of the SELDI–TOF–MS analysis are shown in Table 2.

On the CM10-array chip, a total of 102 different non-redundant protein signals were detected in the whole stimulated saliva samples. From these, 70 were of high intensity and 63 of low molecular weight. In the pellicle layers, a total of 46 proteins were detected on the CM10 array chip, with 18 in the low molecular range.

In the case of the Q10-arrays, a total of 90 non-redundant proteins were analysed in the saliva samples, with 56 of high intensity. Out of these, 54 proteins were of low molecular weight.

In the pellicle layer, 42 different proteins were detected on the Q10 chip, with 13 in the low molecular range.

The combined detection of CM10 and Q10 revealed 90 proteins in the saliva. Out of these, 56 were of high intensity, with 53 of low molecular weight and three of high molecular weight. Futhermore, 39 high-intensity proteins were detected in the combined analysis for all pellicle layers with 16 of low molecular weight.

For three tested composites, significant differences to the controls were detected after oral exposure for 30 min (Table 3). In the case of Venus Diamond, protein *m*/*z* 1634 showed a significant increase (*p* = 0.008). For GrandioSO, proteins *m*/*z* 1634 and *m*/*z* 1678 were of stronger intensity (*p* < 0.05). On Dyract^®^ eXtra, the proteins *m*/*z* 1634 (*p* = 0), *m*/*z* 1661 (*p* = 0.012) and *m*/*z* 1678 (*p* = 0) were significantly increased. The results are listed in Table 3.

For the compomer Dyract^®^ eXtra, significant differences in the proteomic profile were analyzed after 90 min of oral exposure only. Five different proteins showed significant increased values in comparison to the enamel control (Table 3).

Pellicle composition on Venus^®^ Diamond, Estelite Σ Quick, and Dyract^®^ eXtra was significantly different to the enamel pellicle after 120 min of oral exposure.

Additionally, the 120-min pellicle demonstrated a greater diversity with a predomination in anionic proteins (Table 3). The most intense signal compared to the controls was detected for *m*/*z* 2481 on Dyract^®^ eXtra.

Significant differences of the composite pellicle layers were not solitary detected to the enamel control, but they were also among the single materials.

For the 30-min pellicle, significant differences were found between Venus^®^ Diamond and Dyract^®^ eXtra, GrandioSO and Dyract^®^ eXtra, as well as Estelite Σ Quick and GrandioSO (Table 4).

The greatest diversity in the proteomic composition was recorded among all tested materials for the 90-min pellicle.

For the 120-min pellicle, the most significant differences were found between Estelite Σ Quick and GrandioSO (Table 4).

For some detected proteins, a change in the intensity depending on the exposure time was observed for the tested composites. An increase in exposure time from 30 to 120 min caused a rise in the proteins *m*/*z* 1703, *m*/*z* 1938, *m*/*z* 3436, *m*/*z* 3480, *m*/*z* 4567, *m*/*z* 6965, and *m*/*z* 10,846 on the CM10 chips.

In detail, the proteins *m*/*z* 1703 and *m*/*z* 1938 showed elevated numbers only for Venus^®^ Diamond, GrandioSO, and Estelite Σ Quick. Protein m/z 6965 was detected in increased levels on all tested composite surfaces, except on Estelite Σ Quick.

In case of the Q10 chip, a rise in *m*/*z* 1709, *m*/*z* 1938, *m*/*z* 3364, *m*/*z* 3436, and *m*/*z* 5424 was measured. Interestingly, oral exposure up to 120 min caused a decrease in m/z 1661, *m*/*z* 1678, *m*/*z* 1843, *m*/*z* 2045, *m*/*z* 2069, *m*/*z* 2260, and *m*/*z* 2481 on the CM10- and Q10-array chip (Table 4).

In summary, it was recognized that the proteomic profile differs among the tested composite materials. Furthermore, it was found that the occurrence of single proteins in the pellicle was influenced by the oral exposure time. In comparison to the enamel controls, the 30-min pellicle showed changes in the proteomic profile for all tested composites. Except for Estelite Σ Quick, no significant differences were observed. In the case of the 90-min pellicle, significant differences to the enamel control were detected for Dyract^®^ eXtra only, while at the same time, there was the most heterogeneous proteomic composition among all tested composites. For the 120-min pellicle, differences to the enamel control were found for all composites, except for the resin-composite GrandioSO. In the case of the 120-min pellicle, there was also the greatest diversity in detected proteins with significant differences to the controls. Furthermore, a predomination in adsorbed anionic proteins was detected for the composite surfaces.

For protein identification, the database www.uniprot.org (accessed on 23 September 2023) was used. A selection of possible proteins in accordance to their detected mass-to-charge signal is shown in Table 5.

## 3. Discussion

The present in situ study aimed to investigate the proteomic profile of an aquiered salivary pellicle fromened on the resin-based dental composites Venus^®^ Diamond, GrandioSO, Estelite Σ Quick, and Dyract^®^ eXtra.

To the best of our knowledge, SELDI–TOF–MS was not yet applied for analyzing pellicle proteins on resin-based composites.

At first, the formed 120-min salivary pellicle on all composite and enamel surfaces was examined by scanning electron microscopy (SEM). As shown, all composite surfaces were covered by a homogenous pellicle that did not appear different from the layer formed on the enamel controls. Detaied examinations showed that all surface irregularities, which were observed prior to the oral exposure, were leveled out by the established pellicle layers.

In similar studies, an SEM analysis was also applied for the characterization of salivary pellicles. It has been recognized that the surface of an early pellicle is characterized by an uneven and knotted morphology [29,30,31]. In this regard, the 120-min pellicle, which was observed in the present investigation, showed a more homogenious appearrance. Detailed observations of early pellicle layers revealed heterogeneous reticular, meshwork-like structures with pores, and globules in diameters ranging between 20 and 60 nm [31,32]. Similar to the present observations, environmental scanning electon microscopy (ESEM) of a 120-min pellicle showed confluent layers that also covered all dental fine structures with an additional appearance of adherent bacterial cells and lipid droplets [31].

In some of the obtained SEM images, adhearant oral bacteria were also visible in the present investigation. As outlined before, the pellicle provides several different encore points such as glycolipids, fibrinogen, and collagen for various pioneer organisms (e.g., *Streptococcus* spp., *Actinomyces* spp.) to attach [33,34,35]. The interconnection with other oral species will then result in the emerging of a structured three-dimensional dental biofilm [18]. Besides bacterial attachment functions, it was realized that the physicochemical surface properties of dental materials are also altered by the established pellicle. Detailed examinations have shown that certain surface characteristics such as wettability and surface-free energy of the substrate can strongly be changed due to the pellicle formation [3,14,20,36].

In the present investigation, all specimens were also subjected to mechanical (Ra) and optical (Rq) profilometry before oral exposure. For the composite samples, a mean Ra of 70 nm was obtained, while the Rq ranged between 57 and 78 nm. In the case of the enamel controls, smoother surface values were obtained (Ra = 50 nm, Rq = 46 ± 16). In this context, it is interesting that the composition of a salivary pellicle seems to be more complex on rough surfaces, while at the same time, morphologic irregularities are leveled out [18,37,38]. The observed roughness values are in line with findings that have been already discussed for modern nano-hybrid resin composites (Ra between 30 and 130 nm) [39,40,41,42].

Furthermore, in the present investigation, unstimulated whole saliva was collected from five participants and analyzed by SELDI–TOF–MS, too. As shown by the results, 102 different proteins were discovered on the CM10 array chips. Out of these, 67 were of low molecular weight (1.5–25 kDa), and 35 were of high molecular weight (25–300 kDa). On the Q10 chips, a total of 90 different proteins with 57 in the low molecular weight range (1.5–25 kDa) and 33 in the high molecular weight range (25–300 kDa) were detected.

A salivary protein composition by SELDI–TOF–MS has also been investigated by other authors. A similar study found 70 different peaks on CM10 chips (39 in the range of 2–10 kDa, 17 from 10–20 kDa, and 14 from 20–100 kDa) and a total of 108 peaks on the Q10 chips with most peaks in the range of below 10 kDa [43]. These findings are in line with the results of the present investigation, while other studies reported from total numbers that ranged between 70 and >1000 proteins [14,44,45,46].

One major issue of the present study was to investigate the proteomic profile of the salivary pellicle formed on different resin-based composites. By SELDI–TOF–MS, a total of 46 high-intensity proteins on the CM10 chips, with 24 of low molecular weight and 22 of high molecular weight, were detected. On the Q10 chips, a total of 42 proteins with 18 in the low molecular weight range and 24 in the high molecular weight range were found.

There are only a few studies elucidating the proteomic composition of a salivary pellicle formed on resin-based composites. A recent study reported from a diversity of 706 proteins in an initial pellicle layer (3 min) formed on hybrid-composite specimens. From these, an overlap of 181 proteins was reported with a total of 1435 different proteins detected in the saliva of five participants [47]. In another study, 453 non-redundant proteins were detected in a 120-min pellicle formed on the resin-composite Filtelk Z250, while 354 were identified on the enamel controls and 217 in the unstimulated whole saliva of 10 volunteers [14].

In the present study, the total number of detected proteins was found to be rather low. On both the CM10 and Q10 chip, a selection of 90 non-redundant proteins in the saliva and 39 in the composite pellicle were analyzed.

The applied SELDI–TOF–MS methode described in the present study has since been further developed. Modern mass spectroscopic techniques show a high sensitivity, which allows for a more efficient detection of pellicle proteins [4,13,48,49]. In addition, the applied CM10 and Q10 array chips in the present investigation allowed for the detection of only a limited and rather selective number of proteins. Both points can be seen as limitations of this study.

However, in the present investigation, an almost equal distribution between proteins of low molecular weights and high molecular weights was observed. In this context, it was shown that small molecular weight proteins (10 to 20 kDa) are mainly present in early pellicles (3-min pellicle). This could be due to the higher mobility and, hence, higher adsorption velocity of small proteins during the initial phase of pellicle formation [47,50].

In the present study, the detected signals by SELDI–TOF–MS were further assigned to a selection of known proteins (Table 5). The results revealed a rather unselected and diverse proteomic profile. In this context, major pellicle proteins such as amylase, carbonic anhydrase VI, cystatins, histatins, lysozyme, statherin, and proline-rich proteins are often detected [3]. In a previous study, it was revealed that members of the S100 protein family especially show a great affinity to dental-restorative materials [14].

Meanwhile, it was observed that in the case of resin-based composites, the kind of filler particles also seem to significantly influence the adsoption of proteins to the material surface. It was shown that the incorporation of fillers generally increased the abundance of salivary pellicle proteins, and that the addition of inhibitors generally increased cystatins, lysozymes, and mucins regardless of the specific inhibitor used [20,51]. Other authors used SDS–PAGE to investigate salivary pellicle composition and concluded that human salivary low-molecular-weight mucin proline-rich proteins and agglutinin were enriched on a glass ionomer cement compared to a resin composite (TPH Spectrum, Dentsply DeTrey) [20,52]. Composite restorations accumulate more biofilm, experience more secondary decay, and require more frequent replacement. In vivo biodegradation of the adhesive bond at the composite–tooth interface is a major contributor to the cascade of events leading to restoration failure. Binding by proteins, particularly gp340, from the salivary pellicle leads to biofilm attachment, which accelerates degradation of the interfacial bond and demineralization of the tooth by recruiting the pioneer bacterium *Streptococcus mutans* to the surface [53]. In this regard, differences in the pellicle composition of the composite materials were also observed in the present study.

In detail, the proteomic profile was depentent upon the composite material and applied oral exposure time. Proteine absorption differed significanly between the single composite materials. Furthermore, exposure up to 120 min caused elevated numbers especually in *m*/*z* 1703, *m*/*z* 1938, *m*/*z* 3436, *m*/*z* 3480, *m*/*z* 4567, *m*/*z* 6965, and *m*/*z* 10846, while at the same time a decrease in *m*/*z* 1661, *m*/*z* 1678, *m*/*z* 1843, *m*/*z* 2045, *m*/*z* 2069, m/z 2260, and *m*/*z* 2481 was observed.

In this regard, different authors reported that the composition of in situ formed acquired pellicle layers on smooth material surfaces are similar to each another, with only minor differences to the referenced enamel controls [14,20,47].

However, there are still controversial discussions regarding these findings. While some authors reported no changes, others found significant differences in the proteomic profile, too [1,14,15,20]. Overall, it was concluded that pellicle formation is highly influenced by individual parameters and, therefore, differs strongly among the participiants enrolled in those investigations [14,47,50,54].

Detailed observations of in situ formed salivary pellicle on resin-based composites are still rare and, hence, needed. In this study, it was shown that the applied SELDI–TOF–MS method was suitable in detecting pellicle proteins on the surface of resin-based composites. The results of the present study revealed new aspects in regard to the formation of salivary pellicle layers on resin composites. It was relaized that protein composition differed among the observed composite materials. In addition, during the process of maturation, an increase in specific proteiens was recorded, while at the same time, certain proteins decreased in number. Furthermore, for some of the proteins, significant differences to the enamel pellicle were investigated, too.

The results of the present study migth lead to future investiagtions regarding the development of new dental materials with bio-repelling and easy-to-clean surface characteristics.

## 4. Materials and Methods

### 4.1. Recruitment of Participants

For this study, six participants (three female and three male) with a mean age of 23.2 years were consecutively recruited by the Department of Conservative Dentistry and Periodontology, University Hospitals, Jena, Germany. Dental health of each subject was determined by the following clinical criteria: approximate plaque index (≤20%), bleeding on probing (≤20%), maximum probing depth (≤3.5 mm), and decayed–missing–filled teeth index (DMFT ≤ 3).

Participants with a history of general disease, drug, or alcohol abuses, as well as smokers, were excluded from the study. The study was approved by the local Ethical Committee (Friedrich–Schiller–University, Medical Faculty, Jena, Germany; ID: B 3079-03/11) and written informed consent of each participant was provided.

### 4.2. Sample Preparation

For this study, the resin composites Venus^®^ Diamond (Kulzer GmbH, Hanau, Germany), GrandioSO (VOCO GmbH, Cuxhaven, Germany), Estelite Σ Quick (Tokuyama Dental, Altenberge, Germany), and the compomer Dyract^®^ eXtra (Dentsply Sirona, Bensheim, Germany) were used. All composites are chracterized in Table 6.

The materials were applied to rectangular molds (7 mm × 11 mm × 1.5 mm), covered by a glass plate, and polymerized using a calibrated dental light curing unit (bluephase, 1.200 ± 10% mW/cm^2^, Ivoclar Vivadent GmbH, Ellwangen, Germany) for 20 s.

The light-cured specimens were then cleaned twice by an ultrasonic treatment in distilled water for 10 min each and subsequently disinfected with 70% ethanol for 30 min. The finished specimens were afterwards stored separately in distilled water at 8 °C until testing.

As controls, bovine enamel samples were used. Therefore, rectangular enamel specimens (7 mm × 11 mm × 1.5 mm) were prepared from bovine incisors and subsequently surface polished, cleaned three times in an ultrasonic bath for 10 min each, and disinfected in a 70% ethanolic solution (30 min). The bovine enamel samples were then stored separately in distilled water at 8 °C until use.

### 4.3. Scanning Electron Microscopic Examination

All tested composite specimens and the bovine enamel control were observed in their native appearance and also after oral exposure for 120 min using the LEO-1530 scanning electron microscope (Zeiss, Oberkochen, Germany). Representative pictures were captured in 20.000× magnification.

### 4.4. Mechanical and Optical Profilometry

The surface of all tested specimens and the controls was observed by mechanical and also optical 3-D profilometry (White-Light Interferometry). For the mechanical testing, the profilometer Hommel Tester T 1000 (Hommelwerke GmbH, Villingen–Schwenningen, Germany) was used. The data were recorded and documented by Turbo DataWin NT V1.34 (JENOPTIK Industrial Metrology Germany GmbH, Villingen–Schwenningen, Germany). Surface roughness was evaluated for each sample over a distance of 1500 µm at a constant speed of 0.15 mm/s.

The optical profilometry was performed using the white light interferometer Zygo NewView 7300 equipped with a 10× objective lens (single measuring field 698 × 523 µm^2^) and a 50× objective lens (single measuring field 140 × 105 µm^2^). Each measurement was repeated five times.

### 4.5. Fabrication of the Miniplast Splint

**Figure 4:**
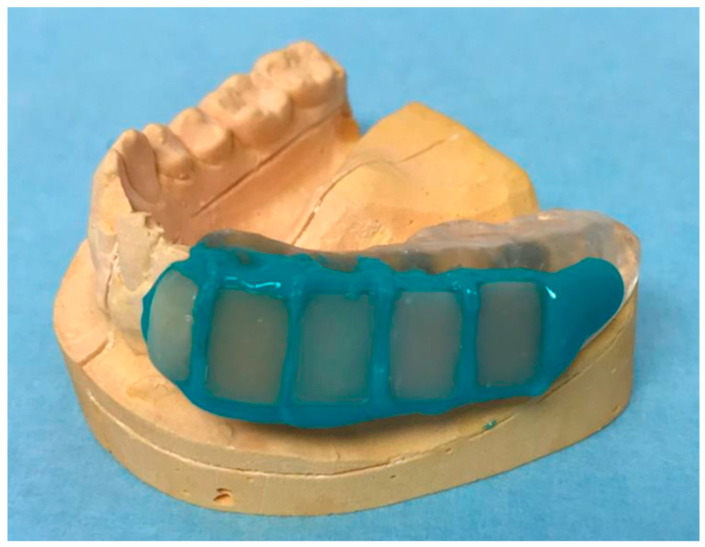
Custom-made removable dental appliance (miniplast splint) with fixed composite specimens and the bovine enamel control. From left to right: bovine enamel control, Dyract^®^ eXtra, Venus^®^ Diamond, Estelite Σ Quick, and GrandioSO.

For oral exposure of all specimens a customized Miniplast splint was used. Therefore, the mandible of each test person was casted using an alginate impression material. After preparation of hard plaster models individual Miniplast splints were fabricated by a thermoplastic deep-drawing process in an Erkoform–3D using an Erkodur film of 2 mm in thickness. The four composite specimens, as well as the bovine control sample, were fixed at the vestibular left side of the Miniplast splint (third quadrant) using the embedding material Aquasil Ultra Flow (Figure 4).

### 4.6. Oral Exposure of the Specimens

One week prior to the study, each test person received a professional dental cleaning, which involved the removal of supragingival hard and soft deposits followed by polishing and a final fluoridation step with Elmex gelee^®^.

At the study day, participants did not eat anything for at least 12 h and were not allowed to brush their teeth in the morning.

The splints were inserted at 8:00 a.m. and remained in the oral cavity for 30 min, 90 min, and 120 min. After each respective exposure time, the test and control specimens were collected, rinsed with aq. dest. for 30 s, and afterwards air-dried.

Subsequently, the surfaces of each specimen that were exposed to the oral environment were whipped using three dental micro brushes (Apply–Tips^®^) moistened with 2% sodium dodecyl sulfate (SDS, 5 µL). A final whip was applied using a dry micro brush. The tips of all four used bushes were removed and transferred to an Eppendorf tube filled with 50 µL of SDS. Subsequently, the specimens were centrifuged for 2 min at 10.000 rpm.

Further, 5 mL of unstimulated saliva were collected from each test person for further investigation of the protein profile and stored until use. In total, four composite speciemens and one enamel control sample were collected from each of the six volunteers at each respective exposure time. Methodontology was partly transferred from [6,15,22,55].

### 4.7. Surface-Enhanced Laser Desorption/Ionization Time of Flight Mass Spectrometry (SELDI–TOF–MS) Analysis

Prior to the SELDI–TOF–MS analysis samples from each specimen were transferred to Q10- and CM10-Array chips. First, the array spots were activated using 5 µL of a respective puffer solution that was applied twice for 5 min each. The Q10-Array puffer contained 0.1 M Tris and 0.02% TritonX-100 (pH = 8.5), while for activation of the CM10-Arrays 0.1 M Sodium acetate (pH = 4) was used.

**Figure 5:**
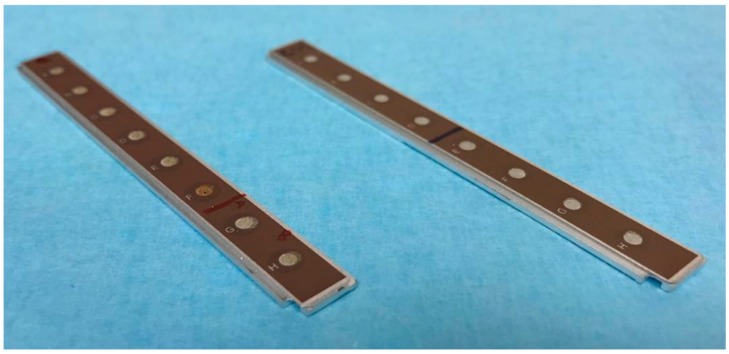
Prepared Q10- and CM10-array chips for SELDI–TOF–MS analysis.

The centrifuged samples of each specimen were mixed in a ratio of 1:1 with the respective puffer solution and subsequently transferred (5 µL) to the activated array spots. After incubation for 90 min in a moistened chamber, each array spot was rinsed three times with the respective puffer solution, followed by another three rinses with aqua dest.. Subsequently, the array chips were passively dried and each spot fixed twice using 0.5 µL of Energy-Absorbing Matrix (EAM: 5 mg sinapric acid, 125 µL 1% trifluoroacetic acid, 125 µL acetonitrile). Prepared arrays are displayed in Figure 5.

For SELDI–TOF–MS analysis, the Ciphergen ProteinChip^®^ Reader System PCS 4000 was used. Each array spot was analyzed four times.

Measurements in the low molecular range (1.500–25.000 kDa) were conducted using a laser intensity of 2200 nJ (Cal2200 fm10), and in the high molecular range (25.000–300.000 kDa), a laser intensity of 3500 nJ (Cal3500 fm50) was chosen. The spectra were then normalized according to three different analysis groups.

Only signals (S) that were five times larger as the noise (N; S/N ≥ 5) were recorded. Furthermore, a minimum threshold of 20% was applied. The determined mass-to-charge ratio was further analyzed by their intensities.

### 4.8. Statistical Analysis

For statistical analysis, SPSS Version 24 was used. Differences in the intensity depending on the material or protein were determined using generalized estimation equations (GEE, link function). Post hoc, *p*-values and confidence intervals were adjusted using the sequential Bonferroni method. The level of significance was set to *p* < 0.05.

## 5. Conclusions

In this study, SELDI-TOF-MS was used to analyze the salivary pellicle layer fromed on various resin-based composites after oral exposure. It was analyzed that pellicle composition varied between individual materials and also differed from the bovine enamel controls. A time-dependent formation was confirmed. The results obtained in the present study could contribute to the development of innovative biorepellent dental materials with easy-to-clean surface properties.

## Figures and Tables

**Table 1:** Mean surface roughness values with standard division obtained by optical and mechanical profilometry (Rq—area-related measurements white light interferometer, Ra—linear measurements mechanical profilometer).

	Rq (nm)		Ra (nm)
	10× Magnification	50× Magnification	
Dyract^®^ eXtra	73 ± 8	78 ± 14	68 ± 8.4
Estelite Σ Quick	95 ± 9	66 ± 13	80 ± 15.8
GrandioSO	92 ± 19	56 ± 17	72 ± 13.0
Venus^®^ Diamond	93 ± 6	78 ± 4	74 ± 11.4
enamel control	71 ± 23	46 ± 16	50 ± 23.5

**Table 2:** Numbers of all non-redundant proteins detected by SELDI–TOF–MS.

	CM10 Array			Q10 Array			CM10 and Q10 Array		
	Intensity		Total	Intensity		Total	Intensity		Total
	I > 1	I < 1		I > 1	I < 1		I > 1	I < 1	
Saliva [Da]									
1.500–25.000	63	4	67	54	3	57	53	3	56
25.000–300.000	7	28	35	2	31	33	3	31	34
			102			90			90
Peliclle [Da]									
1.500–25.000	18	6	24	13	5	18	16	5	21
25.000–300.000	0	22	22	0	24	24	0	18	18
			46			42			39

**Table 3:** Proteins detected by SELDI–TOF–MS on various composite surfaces in comparison to the bovine enamel control. Specimens were exposed to the oral environment for 30, 90, or 120 min.

Proteinn	Dental	Exposure	Mean	Standard	Bonferroni	Array
(*m*/*z*)	Composite	Time [min]	Difference	Error	Sequence	Chip
					(Sequential)	
1634	Venus^®^ Diamond	30	5.194	1.555	0.008	Q10
	GrandioSO		6.682	2.312	0.031	Q10
	Dyract^®^ eXtra		5.209	1.022	0	Q10
1661	Dyract^®^ eXtra	30	8.059	2.485	0.012	Q10
1678	GrandioSO	30	4.92	1.321	0.002	Q10
	Dyract^®^ eXtra		2.877	0.6	0	Q10
1634	Dyract^®^ eXtra	90	7.33	2.720	0.042	Q10
1678	Dyract^®^ eXtra	90	11.631	3.639	0.014	Q10
1843	Dyract^®^ eXtra	90	10.386	3.336	0.018	CM10
2069	Dyract^®^ eXtra	90	17.156	5.829	0.029	CM10
6965	Dyract^®^ eXtra	90	0.676	0.237	0.043	CM10
1634	Venus^®^ Diamond	120	7.798	1.407	0	Q10
	Estelite Σ Quick		10.422	2.931	0.003	Q10
	Dyract^®^ eXtra		7.51	1.439	0	Q10
1661	Venus^®^ Diamond	120	3.063	1.139	0.05	Q10
	Estelite Σ Quick		4.236	0.607	0	Q10
	Dyract^®^ eXtra		1.135	0.39	0.029	Q10
1678	Venus^®^ Diamond	120	10.349	1.718	0	Q10
	Estelite Σ Quick		7.367	1.812	0	CM10
	Dyract^®^ eXtra		9.152	2.044	0	Q10
1843	Venus^®^ Diamond	120	12.829	2.903	0	Q10
	Estelite Σ Quick		7.965	2.385	0.008	CM10
	Dyract^®^ eXtra		15.184	4.619	0.009	Q10
2045	Venus^®^ Diamond	120	16.336	3.589	0	Q10
	Dyract^®^ eXtra		18.686	5.976	0.016	Q10
	Dyract^®^ eXtra		16.746	4.32	0.001	CM10
2069	Venus^®^ Diamond	120	20.723	4.004	0	Q10
	Estelite Σ Quick		14.692	4.186	0.004	CM10
	Dyract^®^ eXtra		19.665	6.362	0.018	CM10
	Dyract^®^ eXtra		23.883	7.313	0.009	Q10

**Table 4:** Significant differences in between the tested dental composites at exposure times of 30, 90, or 120 min.

Composite A	Composite B	Protein (*m*/*z*)	Exposure Time	Mean Difference	Standard Error	Bonferroni Sequence (Sequential)	Array
Venus^®^ Diamond	Dyract^®^ eXtra	1843	30	8.526	1.479	0	CM10
Venus^®^ Diamond	Dyract^®^ eXtra	2069	30	11.999	2.998	0.001	CM10
GrandioSO	Dyract^®^ eXtra	1938	30	1.491	0.372	0.001	Q10
Estelite Σ Quick	GrandioSO	1661	30	7.596	2.315	0.010	CM10
Venus^®^ Diamond	GrandioSO	1634	90	9.306	1.718	0	Q10
Venus^®^ Diamond	GrandioSO	1843	90	13.822	4.78	0.031	CM10
Venus^®^ Diamond	GrandioSO	2260	90	14.779	5.209	0.045	CM10
Venus^®^ Diamond	Estelite Σ Quick	1634	90	8.138	2.574	0.011	Q10
GrandioSO	Estelite Σ Quick	5784	90	2.68	0.944	0.045	CM10
GrandioSO	Dyract^®^ eXtra	3364	90	10.726	3.745	0.038	Q10
Estelite Σ Quick	GrandioSO	1661	90	6.215	1.869	0.009	CM10
Estelite Σ Quick	Dyract^®^ eXtra	3364	90	18.356	5.608	0.011	Q10
Estelite Σ Quick	Dyract^®^ eXtra	3436	90	34.798	9.988	0.005	Q10
Estelite Σ Quick	Dyract^®^ eXtra	3708	90	2.144	0.666	0.013	Q10
Dyract^®^ eXtra	Venus^®^ Diamond	1661	90	11.751	3.954	0.024	Q10
Dyract^®^ eXtra	Venus^®^ Diamond	5424	90	0.572	0.179	0.014	CM10
Dyract^®^ eXtra	GrandioSO	1634	90	10.618	2.827	0.001	Q10
Dyract^®^ eXtra	GrandioSO	1661	90	10.337	2.934	0.004	Q10
Dyract^®^ eXtra	GrandioSO	1843	90	17.881	5.723	0.018	CM10
Dyract^®^ eXtra	GrandioSO	2069	90	27.639	8.461	0.011	CM10
Dyract^®^ eXtra	Estelite Σ Quick	1634	90	9.45	2.264	0	Q10
Dyract^®^ eXtra	Estelite Σ Quick	1661	90	11.441	3.571	0.012	Q10
Dyract^®^ eXtra	Estelite Σ Quick	1678	90	8.661	3.067	0.043	Q10
Estelite Σ Quick	GrandioSO	1634	120	8.445	1.689	0	Q10
Estelite Σ Quick	GrandioSO	1843	120	11.532	3.79	0.019	Q10
Estelite Σ Quick	GrandioSO	1843	120	6.762	1.683	0.001	CM10
Estelite Σ Quick	GrandioSO	2069	120	17.889	5.271	0.006	Q10
Estelite Σ Quick	GrandioSO	2260	120	15.317	5.142	0.023	Q10
Estelite Σ Quick	GrandioSO	2481	120	20.661	6.425	0.01	Q10
Estelite Σ Quick	Dyract^®^ eXtra	1661	120	3.101	0.617	0	Q10

**Table 5:** Identification of selected proteins by their *m*/*z* signal using the database www.uniprot.org, accessed on 23 September 2023.

MW [Da]	Assinged Proteins	UniProt Registration
1678	2′-5′ oligoadenylate synthetase 1 protein	C6EMZ7_HUMAN
	Anaplastic lymphoma kinase	B2MXD8_HUMAN
	Himp	A0A140KRR5_HUMAN
	Protein FAM114A2	E5RIK7_HUMAN
1703	Uncharacterized protein	Q69YS1_HUMA
1938	Ax glycosyltransferase	G9HR99_HUMAN
	NADH dehydrogenase subunit 1	Q5Q8C5_HUMAN
2481	Mitogen-activated protein kinase 10	A0A1W2PNF5_HUMAN
3364	cAMP-regulated phosphoprotein 21	C9J2U3_HUMAN
	M3 muscarinic receptor	Q8NG01_HUMAN
	Mitogen-activated protein kinase 3	J3QS54_HUMAN
	Retina-specific ABC transporter	Q86V62_HUMAN
3480	ABL1 protein	Q86Y36_HUMAN
	Cellular tumor antigen p53	I3L0W9_HUMAN
	ETB1 protein	Q16261_HUMAN
	Glycophorin B	A0A3G2LR13_HUMAN
		A0A346RF30_HUMAN
	Protein yippee-like 3	H3BNP5_HUMAN
3708	Tal-1 product	Q9UE36_HUMAN
	Trimeric intracellular cation channel typ B	X6RGH1_HUMAN
4567	Methyl-CpG-binding domain protein 3	A0A087WVG6_HUMAN
	STK4 protein	Q9BS84_HUMAN
5424	Guanine nucleotide-binding protein G(i) subunit alpha-1	A0A3B3IS42_HUMAN
	Microcephalin	Q6RB59_HUMAN
	Zinc finger protein 385B	C9J0U3_HUMAN
	2-hydroxy-3-oxopropionate reductase	A0A3D2YA96_PSESP
5784	Arf-GAP with coiled-coil, ANK repeat and PH domain-containing protein 2	F8WAU0_HUMAN
	Tryptophan--tRNA ligase, cytoplasmic	G3V2F2_HUMAN
6965	DNA cytosine-5 methyltransferase 2 isoform D	A0A0U1SZ86_HUMAN
	Putative deoxyribonuclease TATDN1	E5RID7_HUMAN
10865	Cytoskeleton-associated protein 2	C9J7Y4_HUMAN
	HCG1748409, isoform CRA_a	A0A024QZ00_HUMAN
	Mediator of RNA polymerase II transcription subunit 15	C9JM58_HUMAN
	MHC class II antigen	A0A1X9I4S0_HUMANA0A6C0TJ11_HUMAN

**Table 6:** Chracterization of the applied composites.

Trade Name	Category	Content		Mean Particle Size (µm)
		Organic Matrix	Inorganic Matrix (Filler)	
Venus^®^ Diamond	nano-hybrid composite	TCD–DI–HEA, UDMA	barium–aluminium–fluoride–glass, nano–SiO_2_–particles	0.005–20
GrandioSO	nano-hybrid composite	Bis–GMA, Bis–EMA, TEGDMA	nano–SiO_2_–particles	0.020–0.040
			glass ceramic	1.0
Estelite Σ Quick	sub-microfiller composite	Bis–GMA, TEGDMA	SiO_2_–ZrO_2_	0.2
Dyract^®^ eXtra	compomer	Bis–EDMA, UDMA, TEGDMA, TMPTMA, TCB	strontium–fluoride–glass	0.8

## Data Availability

Data can be obtained on request from the corresponding author.
